# A systematic screen of conserved *Ralstonia solanacearum* effectors reveals the role of RipAB, a nuclear‐localized effector that suppresses immune responses in potato

**DOI:** 10.1111/mpp.12774

**Published:** 2019-01-09

**Authors:** Xueao Zheng, Xiaojing Li, Bingsen Wang, Dong Cheng, Yanping Li, Wenhao Li, Mengshu Huang, Xiaodan Tan, Guozhen Zhao, Botao Song, Alberto P. Macho, Huilan Chen, Conghua Xie

**Affiliations:** ^1^ Key Laboratory of Potato Biology and Biotechnology, Ministry of Agriculture and Rural Affairs Huazhong Agricultural University Wuhan 430070 China; ^2^ Key Laboratory of Plant Resources, Institute of Botany Chinese Academy of Sciences Beijing 100093 China; ^3^ Key Laboratory of Horticultural Plant Biology, Ministry of Education Huazhong Agricultural University Wuhan 430070 China; ^4^ Shanghai Center for Plant Stress Biology, CAS Center for Excellence in Molecular Plant Sciences, Shanghai Institutes of Biological Sciences Chinese Academy of Sciences Shanghai 201602 China

**Keywords:** type III effector, *Ralstonia solanacearum*, plant immunity, potato, calcium signalling

## Abstract

Both *Solanum tuberosum* and *Ralstonia solanacearum* phylotype IIB originated in South America and share a long‐term co‐evolutionary history. However, our knowledge of potato bacterial wilt pathogenesis is scarce as a result of the technical difficulties of potato plant manipulation. Thus, we established a multiple screening system (virulence screen of effector mutants in potato, growth inhibition of yeast and transient expression in *Nicotiana benthamiana*) of core type III effectors (T3Es) of a major potato pathovar of phylotype IIB, to provide more research perspectives and biological tools. Using this system, we identified four effectors contributing to virulence during potato infection, with two exhibiting multiple phenotypes in two other systems, including RipAB. Further study showed that RipAB is an unknown protein with a nuclear localization signal (NLS). Furthermore, we generated a *ripAB* complementation strain and transgenic *ripAB*‐expressing potato plants, and subsequent virulence assays confirmed that *R. solanacearum* requires RipAB for full virulence. Compared with wild‐type potato, transcriptomic analysis of transgenic *ripAB*‐expressing potato plants showed a significant down‐regulation of Ca^2+^ signalling‐related genes in the enriched Plant–Pathogen Interaction (PPI) gene ontology (GO) term. We further verified that, during infection, RipAB is required for the down‐regulation of four Ca^2+^ sensors, *Stcml5*, *Stcml23*, *Stcml‐cast* and *Stcdpk2*, and a Ca^2+^ transporter, *Stcngc1*. Further evidence showed that the immune‐associated reactive oxygen species (ROS) burst is attenuated in *ripAB* transgenic potato plants. In conclusion, a systematic screen of conserved *R. solanacearum* effectors revealed an important role for RipAB, which interferes with Ca^2+^‐dependent gene expression to promote disease development in potato.

## Introduction

Potato (*Solanum tuberosum L.*) is the third most important food crop worldwide. More than a billion people consume potato, and its production reached 382 million metric tons in 2014 (Kadota *et al*., 2016; McIntyre, 2003). However, potato production and quality are severely constrained by numerous diseases, such as bacterial wilt, caused by *Ralstonia solanacearum* (Champoiseau *et al*., 2009; Wullings *et al*., 1998). The major pathovar infecting potato belongs to phylotype IIB, which is widely distributed worldwide (Fegan *et al*., 2005; Hayward, 2003; Janse, 1996; Seal *et al*., 1999). Phylotype IIB pathogens have adapted to cool temperatures and have migrated worldwide, probably through contaminated potato tubers (Hayward, 1964; Hooker, 1981; Huerta *et al*., 2015; Wang *et al*., 2017; Wicker *et al*., 2012). Even though potato and *R. solanacearum *have a long‐term co‐evolutionary history, very little is known about the interaction between them, as a result of both a lack of resistance materials and technical barriers involving potato genetic manipulation (Chen *et al*., 2013, 2016). New strategies and biological tools are seriously needed for research and disease control.

Type III effectors (T3Es) are key virulence factors of *R. solanacearum* to counteract host immunity (Deslandes and Genin, 2014; Macho and Zipfel, 2015; Peeters *et al*., 2013b), and T3Es from different bacterial pathogens have also been shown to manipulate other cellular functions independent of immunity (Macho, 2016). Therefore, the use of effectors as molecular probes to detect potato immunity would offer a new perspective to study host resistance in potato. To date, this strategy has been broadly used in many studies of plant pathogenesis (Block and Alfano, 2011; Greenberg and Vinatzer, 2003; Kay and Bonas, 2009; Lindeberg *et al*., 2012; Mudgett, 2005; Zhou and Chai, 2008). With the development of genomics, an increasing number of *R. solanacearum* strains have been sequenced, and a plethora of effectors have been identified (Burstein *et al*., 2009, Peeters *et al*., 2013a; Petnickiocwieja *et al*., 2002). Currently, more than 100 effectors have been reported in the *R. solanacearum* species complex (Deslandes and Genin, 2014; Lonjon *et al*., 2018; Peeters *et al*., 2013a). Hence, a larger scale functional screening of effectors is essential for both the provision of more basic information on the effector inventory and acceleration of research on host resistance.

*Ralstonia solanacearum* can infect more than 200 different species of plant. Each individual *R. solanacearum* strain harbours a large effector inventory (60–70) that can respond to various host environments (Genin and Denny, 2012). *Ralstonia solanacearum* may require different combinations of effectors to infect specific hosts. Under these circumstances, the virulence screening of effector mutants is necessary for the study of specific host interactions and to prioritize effector studies amongst a large inventory.

Infection assays using effector mutants provide a direct proof of their contribution to virulence, but the detection of reduced virulence of individual effector mutants is difficult because of functional redundancy (Genin and Denny, 2012; Macho *et al*., 2010; Poueymiro *et al*., 2014). Owing to the conserved biochemical function of Gram‐negative bacterial effectors in eukaryotic environments, heterologous expression in yeast offers an effective and promising tool for whole‐genome functional screening and biochemical studies (Bosis *et al*., 2011; Curak *et al*., 2009). The function of numerous bacterial effectors has been revealed using yeast as a model organism (Fujiwara *et al*., 2016; Mukaihara *et al*., 2016; Salomon *et al*., 2011; Turgeon *et al*., 2009). Transient expression in *Nicotiana benthamiana* is also an ideal system for genetic investigations of plant interactions with pathogens, such as oomycetes, fungi and bacteria (Bombarely *et al*., 2012; Goodin *et al*., 2008; Oh *et al*., 2009; Poueymiro *et al*., 2009). All of these methods combined would provide versatile tools and perspectives to benefit our research.

Compared with *Pseudomonas* and *Xanthomonas*, *Ralstonia* harbours a larger effector repertoire of approximately 110 members (Lonjon *et al*., 2018; Peeters *et al*., 2013a). To date, the biochemical functions of a small number of these effectors and their targets have been described. RipP2 (PopP2) functions as an acetyltransferase that targets WRKY transcription factors (Le Roux *et al*., 2015; Sarris *et al*., 2015). RipAY acts as a γ‐glutamyl cyclotransferase, altering intracellular redox homeostasis *in planta* (Fujiwara *et al*., 2016; Mukaihara *et al*., 2016; Sang *et al*., 2018). RipG family members can interfere with E3 ligase complexes (Angot *et al*., 2006; Remigi *et al*., 2011; Wang *et al*., 2015). RipTPS acts as a trehalose‐6‐phosphate (T6P) synthase, promoting the biosynthesis of T6P, and thus altering the crosstalk between plant metabolism and development (Poueymiro *et al*., 2014). RipAK can suppress the plant hypersensitive response (HR) by the inhibition of catalases (Sun *et al*., 2017). However, the function of most *Ralstonia* effectors is not yet understood.

In this work, we used a multiple screening system, involving the virulence screening of effector mutants in potato, yeast growth inhibition and transient expression in tobacco, to identify *R. solanacearum *virulence effectors. Using this screening system, we identified a highly relevant T3E, RipAB, which is the first reported effector that can suppress the Ca^2+^ signalling pathway at the mRNA level to promote the infection of *R.* *solanacearum* in potato plants.

## Results

### Multiple functional screening reveals T3Es required for *R. solanacearum *virulence

To establish the multiple screening system, we first analysed the effector repertoire of 13 sequenced phylotype IIB strains (UW551, 23_10BR, CFBP1416, CFBP6783, CIP417, IBSBF1503, NCPPB909, NCPPB_282, P673, Po82, UW163, UW179 and UY031) in the *R. solanacearum* T3E database (Peeters *et al*., 2013a). Thirty‐three effectors, which were present more than 10 times in the 13 sequenced strains, were selected as core effectors. Previous studies have shown that 20 and four core effectors are up‐regulated inside plant xylem and root tissues, respectively, in comparison with their expression in rich medium (Jacobs *et al*., 2013, Puigvert *et al*., 2017). This indicates that most of these core effectors are associated with plant infection processes (Table 1). To reveal the virulence contribution of these core effectors, three different functional screenings were performed, including virulence assays of mutants on potato, growth inhibition of yeast and transient expression in *N. benthamiana*.

**Table 1:** The virulence functional screening of *Ralstonia solanacearum* effectors.

Name†	Description‡	Presence in phylotype IIB (%)§	Expression level¶		Yeast growth inhibition††		Cell death in *Nicotiana*‡‡	Disease index in potato§§	
			Stem vs. Medium (log_2_ FC)	Root vs. Medium (log_2_ FC)	30 °C	0.5M NaCl	*N. benthamiana*	Disease index	Significance
ripA2	AWR2	100	2.92	ns	None	None	None	–	–
ripA5_2	AWR5	77	ns	ns	Strong	Strong	–	0.9219 + 0.1197	ns
ripAB	NLS‐harbouring protein	100	27.80	4.27	Weak	Weak	Strong	0.7813 + 0.2016	**
ripAC	LRR domain	92	17.11	3.35	None	None	–	0.9063 + 0.125	ns
ripAD	–	92	3.87	ns	None	None	None	0.8281 + 0.1505	ns
ripAE	Putative acetyltransferase	77	10.09	ns	None	None	None	0.9063 + 0.125	ns
ripAI	–	92	3.51	ns	None	None	None	–	–
ripAJ	–	92	ns	ns	None	None	None	0.9531 + 0.1008	ns
ripAM	–	85	2.07	ns	None	None	None	0.75 + 0.2887	***
ripAN	–	85	3.58	ns	None	Strong	–	0.7188 + 0.2213	****
ripAP	–	77	ns	ns	None	None	None	0.9531 + 0.1008	ns
ripAR	Ubiquitin ligase domain	85	ns	ns	None	None	None	–	–
ripAT	–	77	3.09	ns	None	None	None	–	–
ripAV	–	92	ns	ns	None	Strong	None	–	–
ripAY	Glutamyl cyclotransferase	100	ns	ns	Strong	Strong	None	–	–
ripB	Nucleoside ribohydrolase	85	ns	ns	None	None	–	–	–
ripBH	–	77	3.03	ns	Strong	Strong	Strong	0.7656 + 0.17	**
ripC1	HAD‐like phosphatase	85	9.87	ns	None	None	None	–	–
ripD	–	77	23.66	3.76	None	Weak	None	–	–
ripE1	–	77	ns	ns	None	None	–	–	–
ripE2	–	85	6.40	ns	None	None	None	–	–
ripF1	T3SS translocator	77	23.73	3.94	None	None	None	–	–
ripF2	T3SS translocator	92	2.25	ns	None	None	Weak	0.9531 + 0.1008	ns
ripG2	F‐box LRR GALA2	77	2.30	ns	None	None	None	0.9844 + 0.0625	ns
ripG6	F‐box LRR GALA6	85	ns	ns	None	None	None	0.8906 + 0.1281	ns
ripG7	F‐box LRR GALA7	92	17.54	ns	None	None	None	0.8906 + 0.1281	ns
ripH1	–	77	2.09	ns	None	None	None	0.9219 + 0.1197	ns
ripN	Nudix hydrolase	77	ns	ns	None	None	–	–	–
ripO1	–	92	ns	ns	None	None	None	0.8906 + 0.1281	ns
ripR	–	100	ns	ns	None	None	None	0.9219 + 0.1197	ns
ripTPS	Trehalose phosphate synthase	92	ns	−2.61	None	None	None	0.9688 + 0.08539	ns
ripU	–	100	3.13	ns	None	None	None	–	–
ripV1	Ubiquitin ligase domain	100	2.97	ns	Weak	Strong	Weak	–	–
UW551	–	–			–	–	Weak	0.9531 + 0.1008	

HAD, haloacid dehydrogenase; LRR, leucine‐rich repeat; NLS, nuclear localization signal; ns, not significant; T3SS, type III secretion system.

^†‡^Effector names and functional descriptions are from the type III effector (T3E) database (Peeters *et al*., 2013a).

§The effector presence percentage was calculated from 13 strains in phylotype IIB.

¶The transcriptomic data are from Jacobs *et al*. (2013) and Puigvert *et al*. (2017), and the values are log_2_ FC (fold change).

††Yeast growth inhibition screenings were performed under two conditions (normal conditions and salt stress conditions with 0.5 m NaCl). The images were obtained 2 days after incubation. Two standards (weak/strong) were used to evaluate yeast, as depicted in Fig. S5.

‡‡Effectors developing cell death phenotypes in *Nicotiana benthamiana*. Cell death triggered by effectors in *N. benthamiana* was evaluated at 96 h post‐inoculation (hpi); two standards (weak/strong) were used to evaluate cell death, as depicted in Fig. S6.

§§Virulence screening of *R. solanacearum* UW551 effector mutants in potato. The disease indices were recorded at 14 days post‐inoculation (dpi) [means + standard deviations (SDs), *n* = 4, grey shading indicates *P* < 0.01, one‐way analysis of variance (ANOVA) and Dunnett’s multiple comparisons test). The table cells with grey shading indicate significant differences from the control.

To investigate which effectors are important for *R. solanacearum* potato infection, we generated a collection of effector knockout mutant. Twenty‐four core effectors were successfully mutated in the reference UW551 strain *via* allelic exchange with spectinomycin resistance (Sm^r^) cassettes (Jacobs *et al*., 2013). Compared with the wild‐type (WT) strain, six mutants were discarded because of distinguishable differences in motility assays and growth assays in rich medium (Figs S1 and S2, see Supporting Information). The remaining 18 mutants were subjected to virulence assays using a susceptible potato accession, C9701, as described previously (Chen *et al*., 2013). Compared with the UW551 WT control, four of these effector mutants (*ripAM*, *ripAB*, *ripAN* and *ripBH*) showed a significant reduction in virulence (Table 1; Figs S3 and S4, see Supporting Information). These data indicate that each of these four effectors is required for infection of the susceptible potato accession C9701.

Furthermore, we used a yeast heterologous expression system to investigate the virulence function of the core effectors. We cloned 33 core effectors into a galactose‐inducible vector and transformed them into the yeast strain BY4741. The transformants were grown on both suppression and induction medium under two different conditions (with and without 0.5 m NaCl). The results showed that four effectors, RipA5, RipBH, RipV1 and RipAY, strongly inhibited yeast growth under the two conditions. However, RipAB, RipAN, RipAV and RipD caused a relatively weak growth inhibition (Table 1; Fig. S5). Together, these data indicate that these effectors interfere with basic cellular functions in yeast.

Interestingly, using transient expression in *N. benthamiana,* we observed that four effectors (RipAB, RipV1, RipBH and RipF2) triggered cell death at 96 h post‐inoculation (hpi) (Table 1; Fig. S6, see Supporting Information). Compared with RipV1 and RipF2, RipBH and RipAB triggered a stronger tissue necrosis (Fig. S7, see Supporting Information). As a control for the immune elicitor that induces cell death, we used the *Phytophthora infestans* elicitor INF1 (Du *et al*., 2015). However, unlike cell death triggered by INF1, the cell death triggered by these four effectors seemed to be independent of the *SGT1*‐, *EDS1*‐, *NDR1*‐, *HSP70*‐ and *MEKK2*‐mediated immune signalling pathways, as virus‐induced gene silencing (VIGS) of these genes did not affect the cell death triggered by these effectors (Figs S8 and S9, see Supporting Information). These findings suggest that the cell death triggered by these effectors may be independent of the signalling pathways mediated by these genes.

As RipAB is required for full virulence during potato infection, induces cell death in *N. benthamiana* and inhibits yeast growth, we selected RipAB as our primary research object.

### RipAB is a type III‐secreted effector *in vivo*

**Figure 1:**
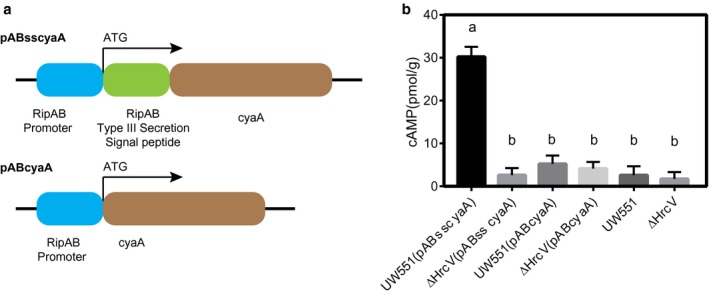
RipAB is a type III‐secreted effector. (a) Schematic representation of the functional domains of the type III secretion system (T3SS) translocation vector pABsscyaA&pABcyaA, including the predicated secretion signal (coding region of 339 bp), the promoter (upstream 200 bp) and *cyaA* gene. (b) RipAB is translocated into plant cells by a T3SS *in vivo*. The cyclic adenosine monophosphate (cAMP) levels were measured at 7 days post‐inoculation (dpi). [Means + standard deviations (SDs), *n* = 3, one‐way analysis of variance (ANOVA) and Tukey’s multiple comparisons test, different letters indicate significant differences, *P* < 0.01).

Previous studies have shown that RipAB is a type III‐secreted effector *in vitro *(Gueneron *et al*., 2000; Lonjon *et al*., 2015). To test the secretion of RipAB during potato infection, an adenylate cyclase (cyaA) assay was performed as described previously (Poueymiro *et al*., 2014), with some modifications. We generated an *R. solanacearum* expression vector (pABsscyaA) using a fragment of 100 amino acids of the RipAB N‐terminal type III secretion signal fused to cyaA protein (Fig. 1a). We then transformed the expression vector and an empty vector (pABcyaA) into both UW551 and the type III secretion system (T3SS) mutant ΔHrcV. If it is translocated by the T3SS, cyaA would significantly increases the concentration of cyclic adenosine monophosphate (cAMP) in the cytoplasm. The concentration of cAMP was monitored at 7 days post‐inoculation (dpi). We observed that the concentration of cAMP in plants inoculated with UW551 (pABsscyaA) was significantly higher than that in plants inoculated with ΔHrcV (pABsscyaA), UW551(pABcyaA), ΔhrcV(pABcyaA), UW551 or ΔHrcV strains (Fig. 1b), indicating that RipAB is secreted by the T3SS, which is consistent with the results of a previous *in vitro *secretome study (Gueneron *et al*., 2000; Lonjon *et al*., 2015).

### RipAB contributes to *R. solanacearum* UW551 infection in potato

**Figure 2:**
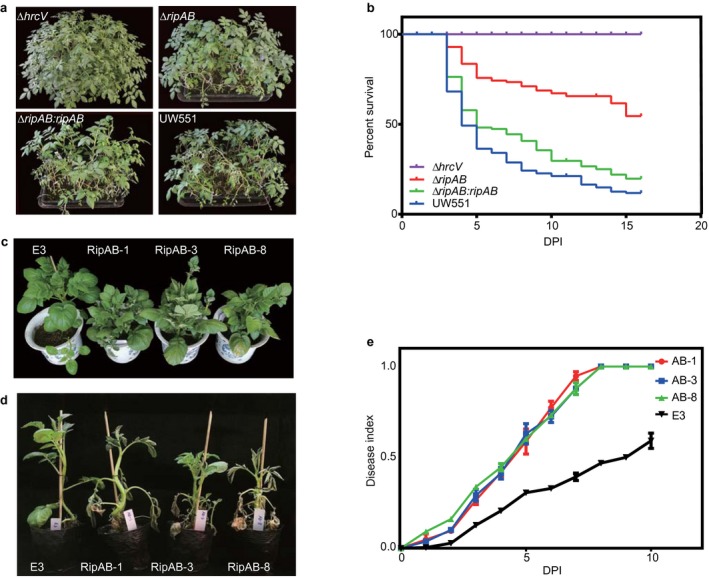
RipAB is required for full virulence in potato. (a) Representative photographs showing the disease symptoms of potato infected by an *hrcV* mutant, a *ripAB* mutant, the complementation strain Δ*ripAB:ripAB* and the wild‐type (WT) strain UW551. The potato root‐drenched infections were performed in the glasshouse to evaluate the virulence of effector mutants. The images shown were obtained at 14 days post‐inoculation (dpi). (b) Survival analysis of potato plants inoculated with *Ralstonia solanacearum* *hrcV* mutant (purple), *ripAB* mutant (red), complementation strain *ΔripAB:ripAB* (green) and WT strain UW551 (blue). One hundred potato plants were inoculated with each individual strain. Statistical analysis was performed via the log‐rank test and the Gehan–Breslow–Wilcoxon test (*P* < 0.01). (c) Representative photographs of RipAB transgenic lines (RipAB‐1, RipAB‐3 and RipAB‐8). The images shown were obtained 6 weeks after planting. (d) Representative images showing the phenotypes of RipAB transgenic potato lines challenged with WT UW551. The images shown were obtained at 14 dpi. (e) Disease indices of *ripAB* transgenic potato lines challenged with WT UW551; included are three transgenic lines, RipAB‐1 (red), RipAB‐3 (blue), RipAB‐8 (green), and potato cv. E3 control (black). Each point represents the disease index of three independent experiments, each comprising 24 plants per treatment.

To confirm the virulence screening data, we performed genetic complementation of the *ΔripAB* mutant. The *ΔripAB:ripAB* complementation strain was constructed via allelic exchange, in which *ripAB* expression is driven from its native promoter, and a kanamycin resistance (Km^r^) cassette was inserted into a permissive chromosomal site, as described previously (Monteiro *et al*., 2012). Consistent with previous screening data, potato infection assays showed that the virulence of the *ΔripAB* strain was significantly reduced (*P* < 0.0001), whereas the *ΔripAB:ripAB *complementation strain recovered the virulence to WT levels (Fig. 2a,b).

To explore this phenomenon further in potato plants, we generated transgenic potato lines expressing *ripAB *from a constitutive 35S promoter. In comparison with other transgenic lines expressing control genes under a 35S promoter, all *ripAB* transgenic lines presented much lower expression of the transgene (Fig. S10, see Supporting Information), suggesting that the plants with high *ripAB* expression were not viable on genetic transformation with the *ripAB* expression construct. *ripAB* transgenic plants exhibited stunted phenotypes (Fig. 2c), suggesting that RipAB alters important plant physiological functions.

Three independent potato transgenic lines (RipAB‐1, RipAB‐3 and RipAB‐8) were subsequently selected and challenged with UW551 (Fig. S11, see Supporting Information). As depicted in Fig. 2d, compared with the untransformed potato cv. E3 control lines, the transgenic lines did not survive for more than 10 dpi with UW551. The three transgenic lines exhibited enhanced disease development, as measured by the disease index (Fig. 2e). Together, these results further confirm that RipAB contributes to UW551 infection in potato.

### RipAB requires a nuclear localization signal (NLS) to trigger cell death in *N. benthamiana*

**Figure 3:**
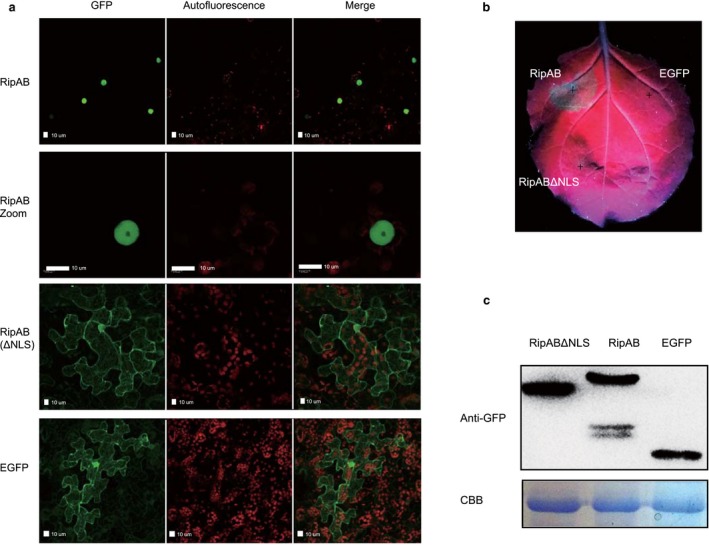
RipAB requires a nuclear localization signal (NLS) to trigger cell death in *Nicotiana benthamiana*. (a) RipAB localized in the nucleoplasm. The confocal images were captured with a Leica SP8 instrument at 36 h after GV3101 (harbouring vector pk7wg2‐RipAB \pk7wg2‐RipABΔNLS\ pk7wg2)‐mediated transient expression in six‐leaf‐stage *N. benthamiana* plants. The white scale bar indicates 10 μm. (b) Mutation of NLS abolished the cell death triggered by RipAB in *N. benthamiana.* pk7wg2 expression plasmids (pk7wg2‐RipAB \pk7wg2‐RipABΔNLS\ pk7wg2) were transformed into *Agrobacterium* GV3101 for transient expression in *N. benthamiana*, and the images shown were obtained at 96 h post‐inoculation (hpi). The experiment was conducted in triplicate. (c) Immunoblots of protein extracts from agroinfiltrated leaves of EGFP‐RipAB, EGFP‐RipAB ΔNLS and enhanced green fluorescent protein (EGFP). The proteins were extracted at 48 hpi via extraction buffer with 1% NP40. GFP antibodies were purchased from Thermo Fisher Scientific. The experiment was conducted in triplicate. The protein loading is indicated by Coomassie Brilliant Blue (CBB) staining.

To predict the molecular function of RipAB, its protein sequence was used to query several protein databases, including the National Center for Biotechnology Information (NCBI), UNIPROT, PHYRE2 and HHpred. However, RipAB did not show any sequence or structural similarity to known proteins. Nevertheless, a nuclear localization signal (NLS) was found in RipAB (Fig. S12, see Supporting Information). To further investigate the function of RipAB, its subcellular localization in plant cells was examined in *N. benthamiana* using confocal microscopy. A construct with enhanced green fluorescent protein (EGFP) fused to the N‐terminus of RipAB was expressed in *N. benthamiana* via *Agrobacterium*‐mediated transient expression. EGFP‐RipAB was observed to localize in the nucleoplasm (Fig. 3a). To determine the relevance of the predicted NLS for RipAB nuclear localization, we generated a RipAB mutant variant with a deletion of the NLS (Fig. S13, see Supporting Information). EGFP‐RipABΔNLS lost the specific nuclear localization (Fig. 3a), indicating that RipAB requires this NLS for its nuclear localization.

As RipAB triggers cell death in *N. benthamiana*, we tested whether the NLS is required for cell death induction. EGFP‐RipAB and EGFP‐RipABΔNLS were transiently expressed in *N. benthamiana* and, as depicted in Fig. 3b,c, EGFP‐RipABΔNLS did not trigger cell death, indicating that the NLS and the nuclear localization of this effector are essential for the induction of cell death.

### RipAB suppresses transcriptional responses associated with calcium signalling

**Figure 4:**
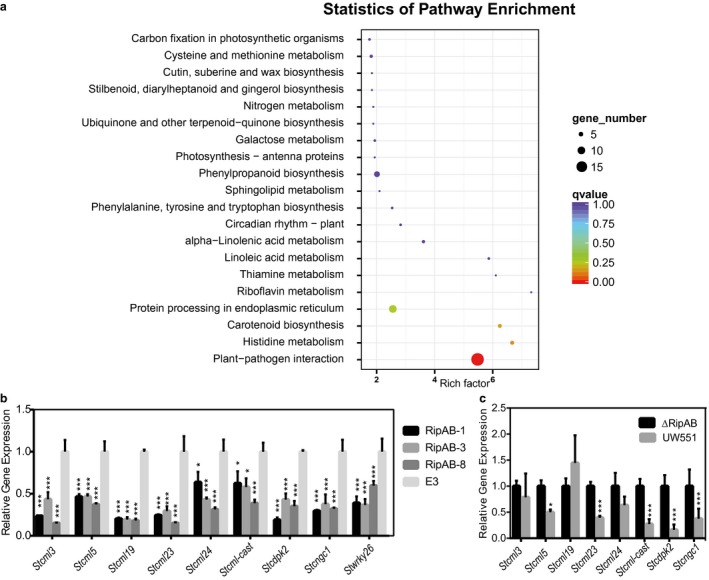
RipAB down‐regulates the calcium signalling pathway at the transcriptional level. (a) Statistics of KEGG pathway enrichment showing that the Plant–Pathogen Interaction (PPI) pathway was the only significantly enriched pathway. Sixteen genes were enriched in the PPI pathway. (b) The relative expression of nine genes involved in the Ca^2+^ pathway was validated by quantitative reverse transcription‐polymerase chain reaction (qRT‐PCR) in transgenic plants. (c) The relative expression of eight genes involved in the Ca^2+^ pathway was validated by qRT‐PCR in potatoes inoculated with mutant UW551 (Δ*ripAB*) and UW551 strains at 24 h post‐inoculation (hpi).

To identify specific plant processes affected by RipAB, we performed a transcriptomic analysis of potato plants expressing *ripAB*. Non‐inoculated roots of three different transgenic lines (RipAB‐1, RipAB‐3 and RipAB‐8) and a control cultivar (E3) were sampled and subjected to RNA sequencing (RNA‐seq) analysis. Four hundred and seventeen differentially expressed genes (DEGs) were identified in all three *ripAB* transgenic lines compared with the E3 control line (Fig. 4a). Notably, most DEGs were down‐regulated (388), whereas only 29 genes were up‐regulated, which indicates that RipAB interferes with the transcription process (Fig. S14, see Supporting Information).

To determine the signalling pathways in which these DEGs are involved, KEGG pathway enrichment analysis was performed. Interestingly, only the Plant Pathogen Interaction (PPI) gene ontology (GO) term was significantly enriched (Fig. 4b). Among all the DEGs, 16 genes were enriched in the PPI GO term, half of which were documented to be involved in Ca^2+^ signalling, including one cyclic nucleotide‐gated ion channel 1 (*Stcngc1*) gene, whose product is a calcium transport channel (Jammes *et al*., 2011), six calmodulin genes, annotated as *Stcml3*, *Stcml5*, *Stcml19*, *Stcml23*, *Stcml24 *and *Stcml-cast*, and one calcium‐dependent protein kinase 2 gene (*Stcdpk2*), whose product is considered to be a calcium sensor that translates calcium signals into phosphorylation signals (Boudsocq and Sheen, 2013). Additional targeted analysis by quantitative reverse transcription‐polymerase chain reaction (qRT‐PCR) confirmed that eight calcium signalling‐related genes were significantly down‐regulated in the three *ripAB*‐expressing transgenic lines (Fig. 4c).

To confirm whether RipAB down‐regulates calcium signalling‐related genes during infection in potato, we investigated gene expression in potato roots after inoculation with UW551 and Δ*ripAB* at 24 hpi. Compared with Δ*ripAB*, infection with UW551 caused a significant down‐regulation of the expression of *Stcml5*, *Stcml23*, *Stcml‐cast*, *Stcdpk2* and *Stcngc1* (Fig. 4d). These data are consistent with the RNA‐seq data from *ripAB* transgenic potato plants, strengthening our conclusion that RipAB specifically and significantly down‐regulates calcium signalling‐related genes during infection.

### RipAB suppresses the pathogen‐associated molecular pattern (PAMP)‐triggered oxidative burst

**Figure 5:**
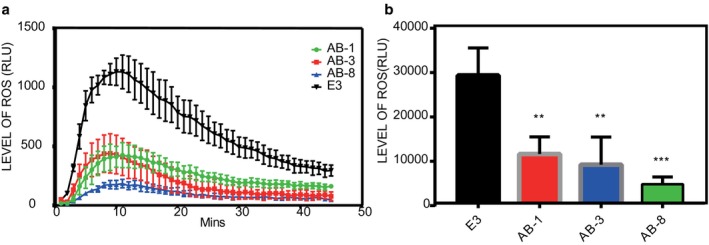
RipAB suppresses the pathogen‐associated molecular pattern (PAMP)‐triggered accumulation of reactive oxygen species (ROS). (a) Time course curve of ROS production in potato leaves, including the three transgenic lines, RipAB‐1 (green), RipAB‐3 (red) and RipAB‐8 (blue), and the potato cultivar E3 control line (black). Each point represents the relative luminescence units (RLU) (mean of three independent experiments), each comprising 24 leaf discs per treatment. The leaf discs were treated with the elicitor flg22 at 100 µm, and flg22‐triggered ROS production was measured as photon counts during a 45‐min period. (b) ROS accumulation during 45 min in potato leaves, including three transgenic lines, RipAB‐1 (red), RipAB‐3 (blue) and RipAB‐8 (green), and the potato cultivar E3 control line (black). [Mean + standard deviation (SD), *n* = 3, ***P < 0.01, one‐way analysis of variance (ANOVA) and Dunnett’s multiple comparisons test.]

As a secondary messenger, Ca^2+^ is required for PAMP‐induced reactive oxygen species (ROS) production mediated by respiratory burst oxidase homologues (RBOHs) (Kadota *et al*., 2015). As such, we speculated that the down‐regulation of genes involved in Ca^2+^‐mediated signal transduction could possibly affect the oxidative burst. Thus, we tested the oxidative burst in transgenic leaf tissues on treatment with the bacterial PAMP flg22, which is the major epitope peptide of bacterial flagellin. In potato plants, the production of ROS reached a peak at 10 min after flg22 treatment. In control plants (E3), a peak value greater than 1000 relative luminescence units (RLU) was reached, whereas the peak value in *ripAB*‐expressing transgenic lines was less than 500 RLU (Fig. 5a). Compared with the E3 control lines, the *ripAB*‐expressing transgenic lines exhibited a strong reduction in total ROS generated during 1 h after flg22 treatment (Fig. 5b), suggesting that the down‐regulation of the Ca^2+^ signalling pathway by RipAB interferes with PAMP‐triggered ROS accumulation.

## Discussion

*Ralstonia solanacearum* can infect more than 200 different plant species, and each individual *R. solanacearum* strain harbours a large effector inventory (60–70) (Genin and Denny, 2012). Thus, *R. solanacearum* might require different combinations of effectors to infect different hosts. Two lines of evidence exist to support this presumption. First, previous studies have shown that a single effector mutant hardly exhibits any virulence reduction in a limited number of hosts (Macho *et al*., 2010; Poueymiro *et al*., 2014). Second, previous studies have shown that *R. solanacearum* RipAY, a glutamyl cyclotransferase, is the only effector that can significantly reduce glutathione in eggplants, whereas the ripAY mutant shows no marked difference in virulence in eggplants (Fujiwara *et al*., 2016). This indicates that RipAY, a non‐redundant effector, is dispensable for virulence on eggplants. In this scenario, the virulence screening of effector mutants is necessary for specific host interaction studies and to prioritize effector studies in a large inventory. To investigate which effectors are important for *R. solanacearum* potato infection, we set up a screen to identify effector mutants with reduced virulence in potato plants. We tested 18 effector mutants, and four exhibited significantly reduced virulence (Fig. S3). Our data suggest that these four effectors contribute to potato infection. However, we must consider that only one accession of susceptible potato was used for this screen, and therefore other relevant effectors could have been overlooked, such as RipAJ and RipG7, which have been shown recently to be under diversifying selection in *R. solanacearum* (Castillo and Agathos, 2018). In the future, additional accessions will be tested in similar screening studies.

Heterologous expression in yeast is a powerful method to identify conserved biochemical functions in effectors that interfere with yeast growth (Popa *et al*., 2016). Via this model system, RipAY was revealed as a glutathione‐degrading enzyme which functions in both yeast and plant cells (Fujiwara *et al*., 2016; Mukaihara *et al*., 2016; Sang *et al*., 2018). In keeping with these reports, we also found that RipAY from UW551 inhibits yeast growth, validating our screening approach for the identification of UW551 effectors that interfere with important functions in eukaryotic cells. In addition, RipA5 was found to inhibit the conserved TOR signalling pathway in yeast (Popa *et al*., 2016). To complement our analysis in yeast cells, we performed transient expression in *N. benthamiana*. We used cell death as a visualized phenotype (Fig. S5).

The development of cell death can be associated with a severe form of immunity, known as the HR. A previous study has shown that manipulation of the Arabidopsis type I metacaspase regulatory module can almost eliminate HR, but this elimination does not lead to increased pathogen proliferation, decoupling cell death from resistance (Coll *et al*., 2010). These findings indicate that HR‐type cell death could be independent of immunity and could be mediated by an independent cell death pathway (Coll *et al*., 2011, 2014; Liu and Levine, 2015). Distinguishing between cell death caused by toxicity and HR is difficult. To identify effectors triggering HR, the corresponding resistance genes (*R* genes) need to be revealed, which, in most cases, requires additional studies and the development of tools in specific host plants. In our research, four effectors caused cell death, which was independent of the usual signalling components required for immunity triggered by INF1. Therefore, we hypothesize that the cell death triggered by these effectors is unlikely to be a result of an HR, and is most likely caused by a toxic effect of their activities in plant cells. First, their conserved effect on both yeast and *N. benthamiana* suggests that these effectors could have a conserved biochemical function in eukaryotic cells. Another line of evidence is that cell death triggered by these effectors is slower than that triggered by INF1. In our study, we observed that INF1‐triggered cell death became evident at 36–42 hpi, whereas these effectors triggered cell death at 72–96 hpi.

We observed that several effectors, such as RipAB, RipBH and RipV1, caused multiple phenotypes in our screening system, suggesting a potential association of these three phenotypes (Figs [Link], [Link], [Link]), and raising the possibility that these effectors have a conserved biochemical function or target a conserved signalling pathway in different organisms. It is possible that these effectors target basic cellular functions; for example, RipAB seems to target host processes associated with the Ca^2+^ signalling pathway, which is essential in all types of biological processes (Cheval *et al*., 2013).

**Figure 6:**
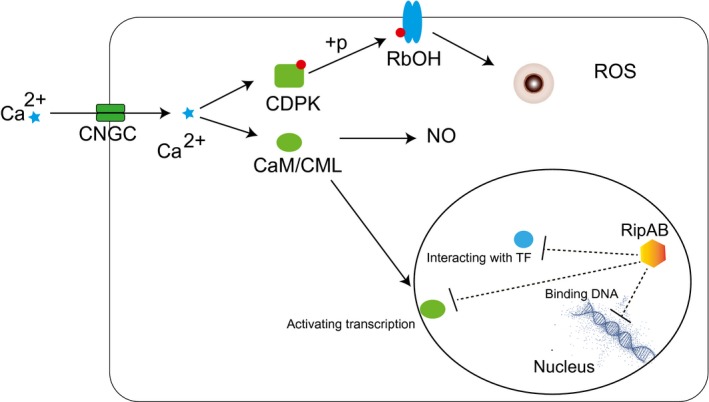
A hypothetical model depicting how RipAB suppresses immunity via the down‐regulation of calcium signalling. RipAB could bind directly to the DNA to impede transcription or could interact with Ca^2+^ signalling regulators or transcription factors (TF). Down‐regulation of the Ca^2+^ transport channel gene *CNGC *would affect Ca^2+ ^influx, and down‐regulation of the calcium sensor would result in the inability of Ca^2+ ^perception, which would lead to the inactivation of downstream immune responses. CaM/CML, Calmodulin; CDPK, calcium‐dependent protein kinase; CNGC, cyclic nucleotide‐gated ion channel; NO, nitric oxide; RBOH, respiratory burst oxidase homologue; ROS, reactive oxygen species.

RipAB was previously named popB (Gueneron *et al*., 2000). However, the function of RipAB is still unknown. Here, we found that RipAB is a nuclear localized protein required for full virulence during potato infection, and our additional molecular analysis showed that RipAB severely down‐regulates Ca^2+^‐related signalling at the transcriptional level (Fig. 4). Plants use secondary messengers, such as Ca^2+^, to perceive stimuli and to adapt to dynamic environments. The alteration of Ca^2+^ levels can translate perceived signals to downstream biological processes (Cheval *et al*., 2013). For instance, plant innate immunity requires Ca^2+^ for the amplification of immune responses (Kadota *et al*., 2015). Cytosolic Ca^2+^ concentration dramatically increases after the perception of PAMPs (Lecourieux *et al*., 2006), resulting in the Ca^2+^ binding of NADPH oxidases (RBOHD), which increases ROS production (Oda *et al*., 2010). Such processes can be completed within 15 min and are amongst the first steps for the activation of PAMP‐triggered immunity (PTI) (Boller and Felix, 2009; Macho and Zipfel, 2014). In keeping with this notion, we found that RipAB down‐regulates the expression of *Stcngc1* a Ca^2+^ transport channel gene, and *Stcdpk2/Stcmls*, Ca^2+^ sensor genes, accompanied by a reduction in PAMP‐triggered ROS production in *ripAB*‐expressing transgenic plants (Figs 4 and 6).

Notably, a previous study has shown that CDPK/CMLs, Ca^2+^ sensors, are essential regulators of plant immunity. Mutation of *cml24* impairs HR mediated by avrRpt2 (Delk *et al*., 2005). In addition, silencing of *cam13* increases the susceptibility to *Tobacco mosaic virus* (TMV) and *R. solanacearum *(Takabatake *et al*., 2007). Overexpression of *cdpk*4/5 in potato can phosphorylate RBOHB, leading to HR‐like cell death (Kobayashi *et al*., 2007). NtCDPK2 autophosphorylation and phosphorylation by an upstream kinase are required for the fine‐tuning of defence signalling cascades (Witte *et al*., 2010). Ca^2+^ is a versatile secondary messenger involved in various developmental and adaptive processes in response to various physiological stimuli (Cheval *et al*., 2013). Therefore, it is important to consider that down‐regulated Ca^2+^ signalling will not only affect immunity, but also globally alter physiological processes, including hormone signalling, metabolism and responses to other environmental stresses, increasing the vulnerability of the plant to the environment.

We propose two hypothetical scenarios for the mode of action of RipAB (Fig. 6). First, RipAB could bind DNA directly to suppress gene expression associated with Ca^2+^ signalling. RipAB is a nuclear protein and requires its NLS to trigger cell death, which suggests that RipAB needs to be localized to the nucleus to function properly. Our RNA‐seq data showed that most DEGs were down‐regulated genes (388), which would support a mechanism whereby RipAB directly binds to DNA to impede their transcription. Because RipAB could specifically suppress the Ca^2+^ signalling pathway at the mRNA level, we speculated that RipAB might mimic a calcium‐related transcription factor to target certain DNA regions. However, the DNA sequence analysis of these down‐regulated calcium‐related genes did not show a clear pattern associated with specific transcription factors. In an alternative scenario, RipAB could interact with Ca^2+^ signalling regulators, such as calmodulins, and this would result in indirect down‐regulation of the DEGs. Both scenarios would lead to the down‐regulation of the Ca^2+^ transport channel gene *CNGC*, which would affect Ca^2+^ influx. Moreover, a reduced expression of calcium sensors would result in the inability of Ca^2+^ perception, which would lead to the inactivation of downstream immune responses (Fig. 6).

## Experimental Procedures

### Strains and vectors

All the strains and vectors used in this study are listed in Table S1 (see Supporting Information).

### Plant materials

Potato plants were grown at 18–25 °C in 24‐cm‐diameter plastic pots in the glasshouse (light intensity ranged from 400 to 1000 µE/m^2^) at the National Centre for Vegetable Improvement (Central China), Huazhong Agricultural University, Wuhan, China.

*Solanum tuberosum *cv. E‐Potato 3 (E3), susceptible to *R. solanacearum*, was used for transgenic manipulation, and *Solanum chacoense *accession C9701 (C9701) was a susceptible wild species used for virulence screening.

*Nicotiana benthamiana* plants were grown in general‐purpose compost under long‐day conditions of 16 h of light at 22 °C in a glasshouse, and the light intensity ranged from 130 to 150 µE/m^2^.

### Yeast manipulation

Yeast cells were cultured in YPD medium (1% yeast extract, 2% peptone, 2% glucose), and yeast transformations were performed via the lithium acetate method (Gietz and Woods, 1998). Thirty‐three effector genes driven by the galactose promoter were inserted into a pYES2 plasmid for expression. The yeast cells were then cultured for 2 days in synthetic dextrose medium (2% glucose, 0.67% yeast nitrogen base without amino acids) + 2% raffinose, after which the cells were diluted to an optical density at 600 nm (OD_600_) of 0.4 in water and plated in repression medium (2% glucose) and induction medium (2% galactose) to monitor cell growth. For yeast protein extraction, the yeast cells were grown for 2 days in SD‐Ura medium + 2% raffinose, followed by dilution to an OD_600_ of 0.4 in liquid induction medium (2% galactose). The cells were subsequently cultured for 12 h before cell lysis.

### Transient expression in *N. benthamiana*

Four‐ to six‐week‐old plants were used for transient expression assays. *Agrobacterium tumefaciens* cultures (strain GV3101) carrying various constructs were cultured overnight in the presence of the appropriate antibiotics at 28 °C. The bacteria were pelleted, centrifuged and resuspended in infiltration buffer [10 mm 2‐(*N*‐morpholino)ethanesulfonic acid (MES; pH 5), 10 mm MgCl_2_ and 0.2 mm acetosyringone], after which their concentration was adjusted to the required OD_600 _of 0.3 for immunoblots and HR imaging, or 0.05 for confocal imaging. After 3 h of incubation, the cultures were infiltrated into the leaves of *N. benthamiana* plants. The HR phenotype of the effectors was captured at 60 hpi.

### Mutagenesis of *R. solanacearum*

To generate mutants of *R. solanacearum*, the target effector genes were replaced with cassettes harbouring Sm^r^. Each sequence included the target gene, and its flanking region amplified from the genome of strain UW551 was inserted into a pEASY‐blunt vector. Reverse amplification of the target gene was used to delete the target gene that generated a linear vector only with the flanking region. An Sm^r^ cassette that contained an independent promoter and terminator was inserted into the linear vector using a ClonExpress II One Step Cloning Kit (Vazyme Biotech, Nanjing, China) to create a pEASY‐target::Sm^r^ vector. The Sm^r^ cassette fragment harbouring the mutant gene flanking region fragments was then amplified and purified for transformation of the *R. solanacearum* strain, as described previously (Jacobs *et al*., 2013). All the mutations were verified by both PCR and RT‐PCR, and all the primers used are listed in Table S2 (see Supporting Information). The complementation strain was constructed via allelic exchange, and *ripAB* with a native promoter and a Km^r^ cassette were inserted into a permissive chromosomal site, as described previously (Monteiro *et al*., 2012).

### Bacterial motility and growth assays

Bacterial motility assays were carried out on semisolid medium containing 1% tryptone and 0.325 g of agar (Boles and McCarter, 2000). Each plate medium was inoculated with 5 µL of bacterial culture at a density of 10^7^ colony‐forming units (CFU)/mL. The radius of cell movement was measured at 3 dpi. The growth assay was conducted with B medium (Hendrick and Sequeira, 1984) that lacked antibiotics. The cultures were inoculated in 50‐mL tubes containing 15 mL of medium to an initial OD_600_ of 0.01, and incubated in a 200‐rpm shaker at 28 °C; bacterial growth was measured spectrophotometrically at OD_600_.

### cAMP assays

The cyaA assay was performed as described previously, with some modifications (Poueymiro *et al*., 2014). Five microlitres of bacterial culture at a density of 10^7^ CFU/mL of strain UW551 and its derivatives harbouring pABsscyaA or pABcyaA plasmids were infiltrated into cut surfaces of potato tubers. The tissues were sampled at 7 days after injection. cAMP levels were monitored with a cAMP enzyme immunoassay kit (New East Biosciences, Malvern, PA, USA) according to the manufacturer’s instructions.

### *Ralstonia solanacearum* infection assays in potato

Two different infection methods were applied in this research.

*In vitro* potato tests were used for mutant screening as described by Chen *et al.* (2013), with some modifications. The concentration of inoculated bacteria was adjusted to 10^7^ CFU/mL. Four replicates of 20 plants were investigated for their disease index at 14 dpi. Accession C9701, used for the virulence assays *in vitro*, was cultivated 3 weeks before inoculation. The disease index was recorded each day after inoculation.

The soil drench method was used for the transgenic lines and complementation virulence assays. With respect to the infection of the transgenic cultivar E3, 24 plants were used for the investigation of the disease index; three replicates were tested. The disease index was recorded each day after inoculation. For the effector complementation virulence assays, 100 plants were used for the investigation of the survival rates. The surviving plants were recorded each day after inoculation, and the concentration of inoculated bacteria was adjusted to 10^7^ CFU/mL.

### Confocal laser scanning microscopy

Confocal images of leaf cells expressing the protein fusions at 36 h after transient expression in six‐leaf‐stage *N. benthamiana* plants were captured with a Leica SP8 (Leica, Wetzlar, Germany) instrument. A wavelength of 488 nm was used for GFP excitation, and the wavelength (500–525 nm) of the emission signal was obtained for the GFP channel. A wavelength of 558 nm was used for chloroplast fluorescence excitation, and emission wavelengths (690–720 nm) were obtained.

### Immunoblot analysis

Samples were collected and ground in liquid nitrogen. Lysis buffer was then used as described previously (Kadota *et al*., 2016). The proteins were separated by 10% sodium dodecylsulfate‐polyacrylamide gel electrophoresis (SDS‐PAGE), transferred to a polyvinylidene fluoride (PVDF) membrane (Biorad, Hercules, CA, USA) and then measured via an ECL plus (Biorad) device. The anti‐GFP and horseradish peroxidase‐conjugated secondary antibodies used in this research were purchased from Thermo Fisher Scientific, Shanghai, China.

### Potato genetic transformation

The *ripAB *gene driven by P35S was transformed into potato cultivar E3 via *A. tumefaciens *(LBA4404)‐mediated transformation, as described previously (Tian *et al*., 2003). The regenerated plants were identified by semiquantitative PCR.

### Transcriptome analysis

In this study, root samples of 4‐week‐old *in vitro* potatoes were harvested. Taking untransformed potato cultivar E3 as a control, we sampled three *ripAB* transgenic lines to constitute three biological replicates. The total RNA was extracted with an RNA extraction kit (Zoman Biotechnology, Beijing, China) according to the manufacturer’s instructions. RNA‐seq was performed by Biomarker Technologies (Beijing, China) with a HiSeq‐xTEN (Illumina, San Diego, CA, USA) system. The data were analysed by the BMK Cloud server. The gene expression levels were quantified by the fragments per kilobase of transcript per million fragments mapped (FPKM) method (Trapnell *et al*., 2010). A value of |log_2_(fold change)| ≥ 1 [false discovery rate (FDR) < 0.01] was set as the threshold for DEGs. GO enrichment analysis of the DEGs was implemented by the GOseq R package (Ashburner *et al*., 2000), and the enrichment of the DEGs in the KEGG pathways was analysed via KOBAS software (Mao *et al*., 2005). Raw sequencing data are available in the Sequence Read Archive under the accession code: PRJNA500722.

### qRT‐PCR

Total RNA was extracted using a Plant RNA kit (Biotek, Winooski, VT, USA) in accordance with the manufacturer’s instructions. First‐strand cDNA was synthesized using an iScript cDNA synthesis kit (Biorad). qRT‐PCR was performed using SYBR Green Supermix (Biorad) and a CFX96 Real Time system (Biorad), and the qRT‐PCR data were analysed as described previously (Livak and Schmittgen, 2001). The potato gene *ef1α* (GenBank accession: AB061263) was used as an internal reference gene (Nicot *et al*., 2005). The primer sets used for qRT‐PCR analysis are listed in Table S2.

### ROS assays

Oxidative bursts were measured as described previously (Gimenez‐Ibanez *et al*., 2009; Sang and Macho, 2017), with some modifications. ROS were elicited with 100 nm flg22, and luminescence was measured during a 45‐min period using a microplate luminescence reader. The luminescence was recorded in each well for 400 ms each minute. The data obtained from the ROS burst assays were analysed and represented in two different ways: the RLU produced per minute (the kinetics of ROS production) and the total cumulative values of RLU for each sample (the total ROS production).

### VIGS assays

VIGS assays were performed as described by Liu *et al*. (2005). The primers used are listed in Table S2.

## Supporting information


**Fig. S1  **Bacterial motility of effector mutants. Bacterial motility was assayed on semisolid medium. Each plate medium was inoculated with 5 µL of bacterial culture at a density of 10^7^ colony‐forming units (CFU)/mL. The radius of cell movement was collected at 3 days post‐inoculation (dpi). [Mean + standard deviation (SD), *n* = 3, ***P < 0.01, one‐way analysis of variance (ANOVA) and Dunnett’s multiple comparisons test.]

**Fig. S2  **Bacterial growth of effector mutants. Bacterial growth was assayed with B medium (Hendrick and Sequeira, 1984) that lacked antibiotics. The optical density at 600 nm were monitored at 2 days post‐inoculation (dpi). [Mean + standard deviation (SD), *n* = 3, ***P < 0.01, one‐way analysis of variance (ANOVA) and Dunnett’s multiple comparisons test.]

**Fig. S3  **Virulence screening of *Ralstonia solanacearum* UW551 effector mutants in potato. The potato unwounded root infection *in vitro* test was used, and the disease grade was determined at 14 days post‐inoculation (dpi). Twenty‐four plants per test were inoculated with strains at a density of 10^7^ colony‐forming units (CFU)/mL. [Mean + standard deviation (SD), *n* = 4, ***P < 0.01, one‐way analysis of variance (ANOVA) and Dunnett’s multiple comparisons test.]

**Fig. S4  **Bacterial growth curve of effector mutants. Bacterial growth was assayed with B medium (Hendrick and Sequeira, 1984) that lacked antibiotics. The (optical density at 600 nm) of *ΔripAB*, *ΔripV1*, *ΔripBH*, *ΔripF2 *and UW551 were monitored spectrophotometrically during a 60‐h period.

**Fig. S5** *Ralstonia solanacearum* UW551 effectors interfere with yeast growth under two conditions. PYES‐nta plasmids fused with effector proteins were transformed into yeast BY4741 and then subjected to synthetic dextrose medium comprising different carbon sources, such as galactose (induction) or glucose (suppression). The yeast growth inhibition screening was performed under two conditions (normal conditions and salt stress conditions with 0.5 m NaCl).

**Fig. S6  **Representative graphs of four effectors developing a cell death phenotype in *Nicotiana benthamiana*. The expression plasmids PH7C10.0 with effectors were transformed into *Agrobacterium* GV3101 for transient expression in *N. benthamiana*, and images were taken at 96 h post‐inoculation (hpi) (*n* = 4).

**Fig. S7  **Average inoculations causing the development of cell death phenotypes for RipAB, RipBH and RipV1. Thirty inoculations were performed with individual effectors. The number of dead cells was counted at 96 h post‐inoculation (hpi). [Mean + standard deviation (SD), *n* = 3, letters indicate significant differences *P* < 0.01, one‐way analysis of variance (ANOVA) and Tukey’s multiple comparisons test.]

**Fig. S8  **Average inoculations causing the development of cell death phenotypes for RipAB, RipBH and RipV1 with respect to virus‐induced gene silencing (VIGS) of plant immune response‐related genes (*NbSGT1*, *NbEDS1*, *bNDR1*, *NbHSP70 *and *NbMEKK2*). Agroinfiltration was performed with *Nicotiana benthamiana *plants at 3 weeks after gene silencing. Thirty inoculations were performed with individual effectors in each gene‐silenced plant. The number of dead cells was counted at 96 h post‐inoculation (hpi). [Mean + standard deviation (SD), *n* = 4, ***P < 0.001, one‐way analysis of variance (ANOVA) and Dunnett’s multiple comparisons test.]

**Fig. S9  **Relative gene expression in plants subjected to virus‐induced gene silencing (VIGS). Plant leaves were sampled at 3 weeks after VIGS. [Means + standard deviations (SDs), *n* = 3, ***P < 0.01, one‐way analysis of variance (ANOVA) and Dunnett’s multiple comparisons test.]

**Fig. S10  **Quantitative reverse transcription‐polymerase chain reaction (qRT‐PCR) of *RipAB *and *GUS* transgenic potato plants. Four‐week‐old potato leaves were sampled. [Means + standard deviations (SDs), *n* = 3].

**Fig. S11  **Semi‐quantitative polymerase chain reaction (PCR) of regenerated *RipAB* transgenic potato plants. Seven transgenic plants of RipAB were sampled and subjected to semiquantative PCR.

**Fig. S12  **Prediction of nuclear localization signals (NLSs). The green letters indicate NLSs.

**Fig. S13  **Schematic representation of the nuclear localization signal (NLS) of RipAB.

**Fig. S14  **Heat map showing the expression patterns of RipAB transgenic lines and cv. E3. The hierarchical clustering is shown of 417 genes identified as differentially expressed in the comparison between RipAB transgenic lines and cv. E3.

**Table S1  **Strains and plasmids.

**Table S2  **List of primers used for quantitative polymerase chain reaction (qPCR) analysis and mutant construction.
