# Supplementary figures and images for: A systematic screen of conserved Ralstonia solanacearum effectors reveals the role of RipAB, a nuclear‐localized effector that suppresses immune responses in potato
Source: Mol Plant Pathol. 2019 Jan 9;20(4):547–61. doi: 10.1111/mpp.12774 (PMC6637881; doi:10.1111/mpp.12774)

S1 Fig.

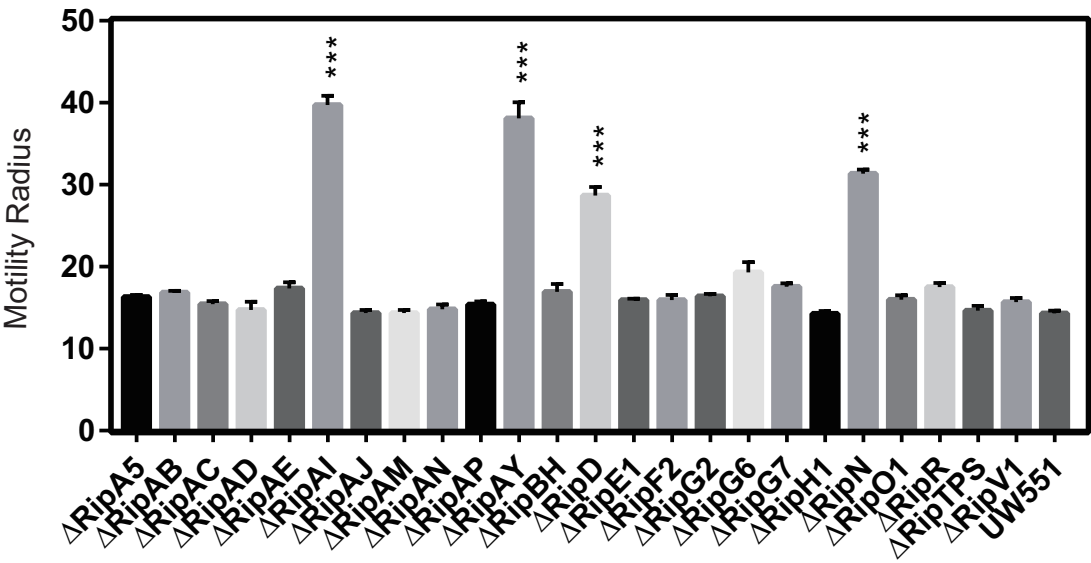

Supplement: Supplementary file 1 — Fig. S1 Bacterial motility of effector mutants. Bacterial motility was assayed on semisolid medium. Each plate medium was inoculated with 5 µL of bacterial culture at a density of 107 colony‐forming units (CFU)/mL. The radius of cell movement was collected at 3 days post‐inoculation (dpi). [Mean + standard deviation (SD), n = 3, ***P < 0.01, one‐way analysis of variance (ANOVA) and Dunnett’s multiple comparisons test.] [file MPP-20-547-s001.pdf]

S2 Fig.

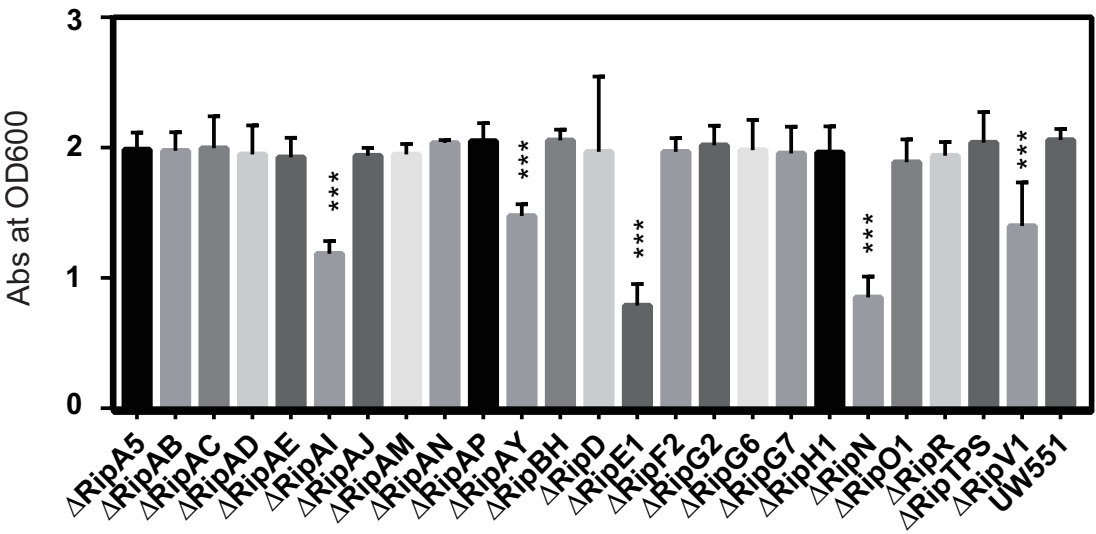

Supplement: Supplementary file 2 — Fig. S2 Bacterial growth of effector mutants. Bacterial growth was assayed with B medium (Hendrick and Sequeira, 1984) that lacked antibiotics. The optical density at 600 nm were monitored at 2 days post‐inoculation (dpi). [Mean + standard deviation (SD), n = 3, ***P < 0.01, one‐way analysis of variance (ANOVA) and Dunnett’s multiple comparisons test.] [file MPP-20-547-s002.pdf]

S3 Fig.

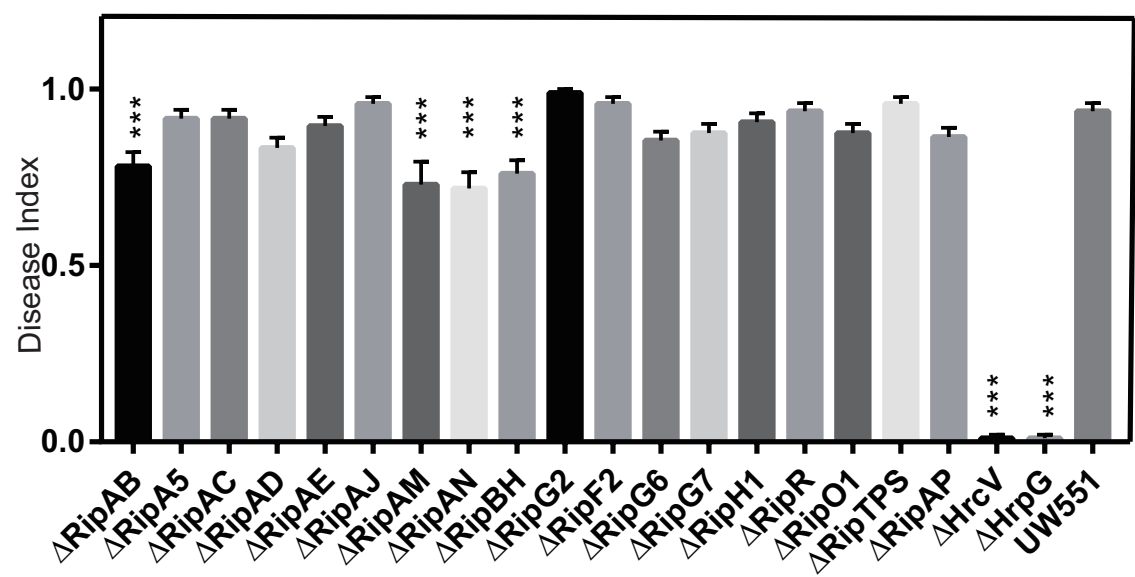

Supplement: Supplementary file 3 — Fig. S3 Virulence screening of Ralstonia solanacearum UW551 effector mutants in potato. The potato unwounded root infection in vitro test was used, and the disease grade was determined at 14 days post‐inoculation (dpi). Twenty‐four plants per test were inoculated with strains at a density of 107 colony‐forming units (CFU)/mL. [Mean + standard deviation (SD), n = 4, ***P < 0.01, one‐way analysis of variance (ANOVA) and Dunnett’s multiple comparisons test.] [file MPP-20-547-s003.pdf]

S4 Fig.

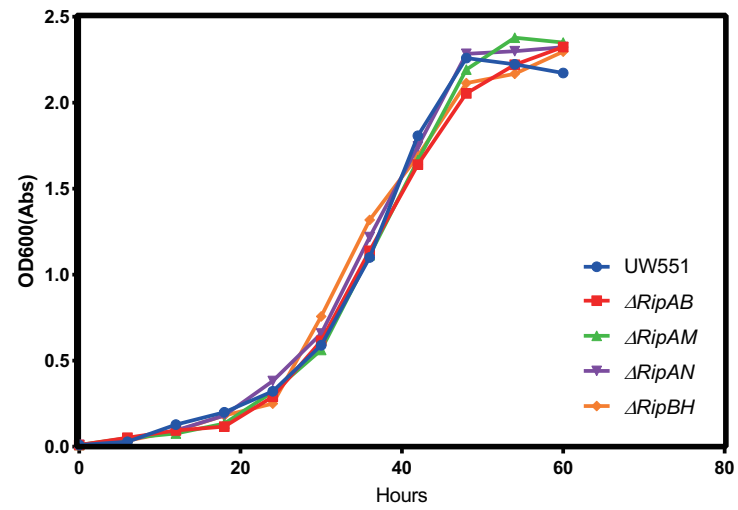

Supplement: Supplementary file 4 — Fig. S4 Bacterial growth curve of effector mutants. Bacterial growth was assayed with B medium (Hendrick and Sequeira, 1984) that lacked antibiotics. The (optical density at 600 nm) of ΔripAB, ΔripV1, ΔripBH, ΔripF2 and UW551 were monitored spectrophotometrically during a 60‐h period. [file MPP-20-547-s004.pdf]

S5 Fig.

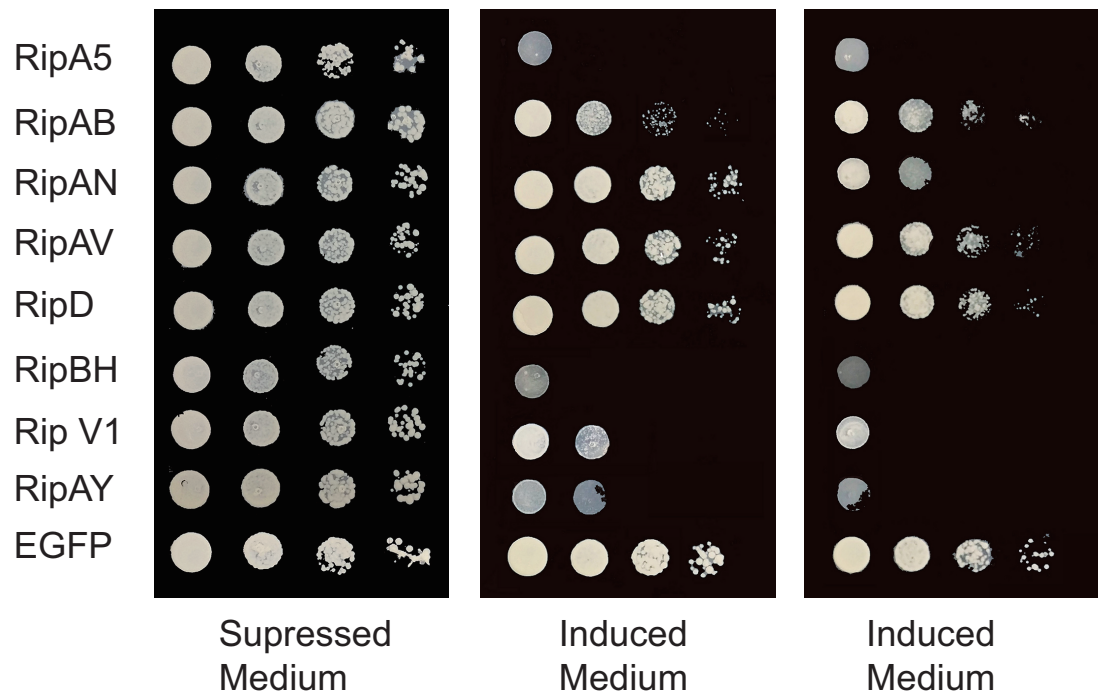

Supplement: Supplementary file 5 — Fig. S5 Ralstonia solanacearum UW551 effectors interfere with yeast growth under two conditions. PYES‐nta plasmids fused with effector proteins were transformed into yeast BY4741 and then subjected to synthetic dextrose medium comprising different carbon sources, such as galactose (induction) or glucose (suppression). The yeast growth inhibition screening was performed under two conditions (normal conditions and salt stress conditions with 0.5 m NaCl). [file MPP-20-547-s005.pdf]

S6 Fig.

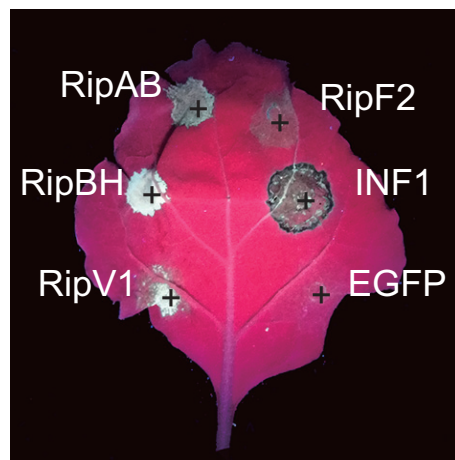

*Nicotiana benthamiana*

Supplement: Supplementary file 6 — Fig. S6 Representative graphs of four effectors developing a cell death phenotype in Nicotiana benthamiana. The expression plasmids PH7C10.0 with effectors were transformed into Agrobacterium GV3101 for transient expression in N. benthamiana, and images were taken at 96 h post‐inoculation (hpi) (n = 4). [file MPP-20-547-s006.pdf]

S7 Fig.

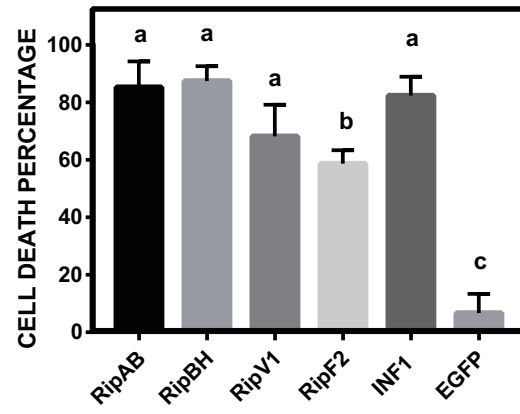

Supplement: Supplementary file 7 — Fig. S7 Average inoculations causing the development of cell death phenotypes for RipAB, RipBH and RipV1. Thirty inoculations were performed with individual effectors. The number of dead cells was counted at 96 h post‐inoculation (hpi). [Mean + standard deviation (SD), n = 3, letters indicate significant differences P < 0.01, one‐way analysis of variance (ANOVA) and Tukey’s multiple comparisons test.] [file MPP-20-547-s007.pdf]

S8 Fig.

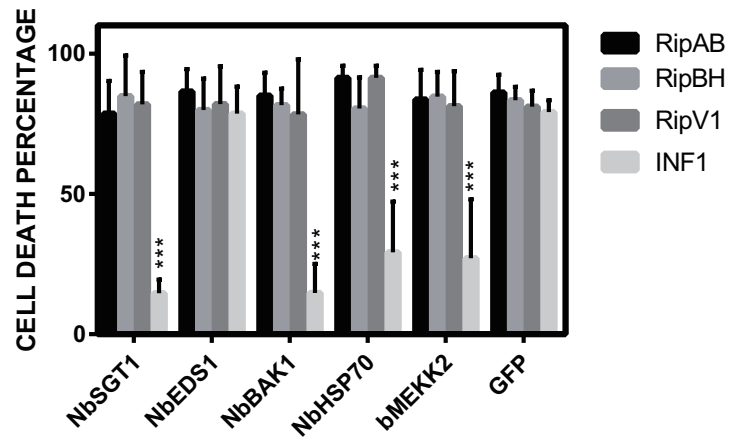

Supplement: Supplementary file 8 — Fig. S8 Average inoculations causing the development of cell death phenotypes for RipAB, RipBH and RipV1 with respect to virus‐induced gene silencing (VIGS) of plant immune response‐related genes (NbSGT1, NbEDS1, bNDR1, NbHSP70 and NbMEKK2). Agroinfiltration was performed with Nicotiana benthamiana plants at 3 weeks after gene silencing. Thirty inoculations were performed with individual effectors in each gene‐silenced plant. The number of dead cells was counted at 96 h post‐inoculation (hpi). [Mean + standard deviation (SD), n = 4, ***P < 0.001, one‐way analysis of variance (ANOVA) and Dunnett’s multiple comparisons test.] [file MPP-20-547-s008.pdf]

S9 Fig.

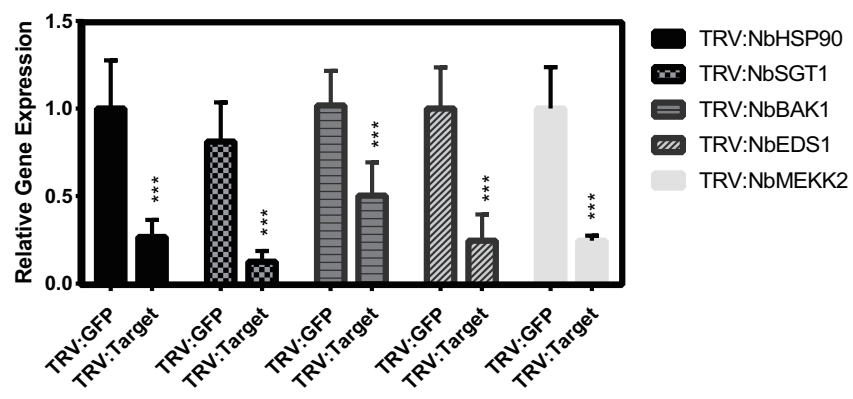

Supplement: Supplementary file 9 — Fig. S9 Relative gene expression in plants subjected to virus‐induced gene silencing (VIGS). Plant leaves were sampled at 3 weeks after VIGS. [Means + standard deviations (SDs), n = 3, ***P < 0.01, one‐way analysis of variance (ANOVA) and Dunnett’s multiple comparisons test.] [file MPP-20-547-s009.pdf]

S10 Fig.

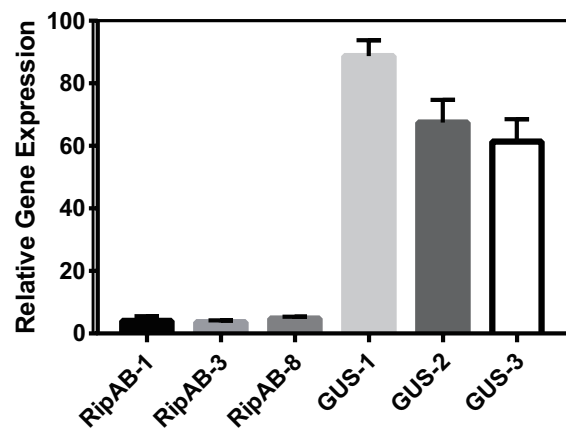

Supplement: Supplementary file 10 — Fig. S10 Quantitative reverse transcription‐polymerase chain reaction (qRT‐PCR) of RipAB and GUS transgenic potato plants. Four‐week‐old potato leaves were sampled. [Means + standard deviations (SDs), n = 3]. [file MPP-20-547-s010.pdf]

S11 Fig.

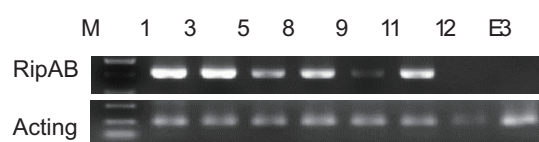

Supplement: Supplementary file 11 — Fig. S11 Semi‐quantitative polymerase chain reaction (PCR) of regenerated RipAB transgenic potato plants. Seven transgenic plants of RipAB were sampled and subjected to semiquantative PCR. [file MPP-20-547-s011.pdf]

S13 Fig.

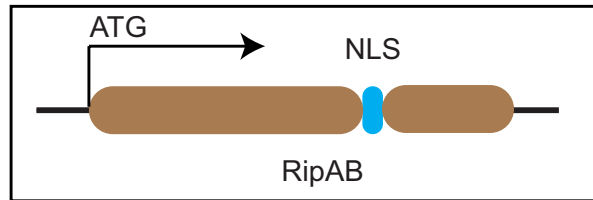

Supplement: Supplementary file 13 — Fig. S13 Schematic representation of the nuclear localization signal (NLS) of RipAB. [file MPP-20-547-s013.pdf]

S14 Fig.

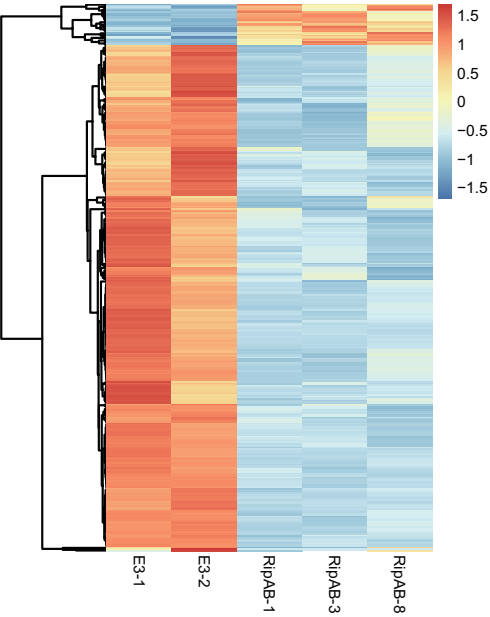

Supplement: Supplementary file 14 — Fig. S14 Heat map showing the expression patterns of RipAB transgenic lines and cv. E3. The hierarchical clustering is shown of 417 genes identified as differentially expressed in the comparison between RipAB transgenic lines and cv. E3. [file MPP-20-547-s014.pdf]
