# Supplementary material for: A systematic screen of conserved Ralstonia solanacearum effectors reveals the role of RipAB, a nuclear‐localized effector that suppresses immune responses in potato
Source: Mol Plant Pathol. 2019 Jan 9;20(4):547–61. doi: 10.1111/mpp.12774 (PMC6637881; doi:10.1111/mpp.12774)
Supplement: Supplementary file 12 — Fig. S12 Prediction of nuclear localization signals (NLSs). The green letters indicate NLSs. [file MPP-20-547-s012.pdf]

S12 Fig.

```
1    MSHSKIRSGGSSGGIGNDLPSTKVPVPSTPTQQPSQL   37
38   NDLLNRGVGNAMNNAGFGSKTSQTWNPDNPIVSL      71
72   KSRSSSGRKPDTGGDTKPESTSGGKRKRDEETDPNN  107
108  EADGKKKKKKRDDEDNASQNGAGSPTGQSGSTPE    141
142  DALMNIVLQRTIQRQTQTRQKMQEAMKIKDEDD*    174
```
